# Suicide Prevention for Older Adults in Residential Communities: Implications for Policy and Practice

**DOI:** 10.1371/journal.pmed.1000254

**Published:** 2010-05-18

**Authors:** Carol A. Podgorski, Linda Langford, Jane L. Pearson, Yeates Conwell

**Affiliations:** 1 University of Rochester Department of Psychiatry, Rochester, New York, United States of America; 2 Institute for the Family, University of Rochester Department of Psychiatry, Rochester, New York, United States of America; 3 Suicide Prevention Resource Center, Newton, Massachusetts, United States of America; 4 Division of Services and Intervention Research, National Institute of Mental Health, Bethesda, Maryland, United States of America; 5 Center for the Study and Prevention of Suicide, University of Rochester Department of Psychiatry, Rochester, New York, United States of America

## Abstract

Examining an often under-appreciated area, Carol Podgorski and colleagues discuss the suicide risk and opportunities for suicide prevention in seniors' residential communities.

## Suicide Risk in Senior Living Communities

Whether by choice or necessity, more older adults are now living in congregate residential settings. About 23% of the 36 million adults over 65 experience relocation [1]. While residences designed to accommodate lifestyle preferences are appealing [2], the underlying reasons (e.g., illness, decline in physical function, loss of a spouse or caregiver) that precipitate moving into a residential home, as well as the ensuing adjustment process, are often stressful. While a move can represent a positive change, all moves involve some degree of loss. These adjustments, coupled with an array of risk factors commonly found among seniors—such as depression, hopelessness, and functional impairment—can result in suicidal behaviors [3]. Despite the growing demand and resultant proliferation of senior housing options, the systems that serve these populations are seemingly unprepared to address suicidal behavior.

Although our understanding of the epidemiology of suicide in older adults is growing, little is known about suicide in senior living settings. The positive perceptions of these settings—such as aging in place, maximizing independence, and promoting safety—along with the high satisfaction rates reported by residents tend to overshadow acknowledgments of distress [4]. Thus policies and best practices regarding suicide prevention in these settings are sparse. In this paper we summarize what is known about suicide risk and suggest opportunities for suicide prevention in senior living communities.

### Residential Communities Defined

Residential communities include independent living communities (ILCs), assisted living facilities (ALFs), long-term care facilities (LTCFs), and continuing care retirement communities, which encompass all three. ILCs, sometimes referred to as retirement or senior living communities, are designed for adults who are able to live independently and desire interaction with peers. ILCs do not provide health care and, hence, are not regulated.

Definitions of assisted living vary by state, accrediting bodies, and providers. Most ALFs include 24-hour supervision, housekeeping, meal preparation, and assistance with activities of daily living (ADLs). Many embrace a philosophy that includes meeting a resident's needs while maximizing independence, privacy, autonomy, and dignity; minimizing the need for relocation; and providing a homelike environment [5]. There are currently over 36,000 ALFs in the United States serving more than one million seniors [6]. In 2000 the average age in ALFs was 85, 79% of ALF residents were female, 99% were white, residents required assistance with an average of 2.3 activities of daily living, 52% had cognitive impairment [5], and 86% paid privately [6].

In 2000 over 1.5 million (4.5%) Americans over age 65 lived in LTCFs, also known as nursing homes (NHs), where care is supervised by licensed nurses [7]. The population was 70% female, 75% of residents were ≥75 years old, the median age was 83.2, 84% were white, and residents required assistance with an average of 3.8 activities of daily living [7].

### Similarities across Settings

While populations across ILCs, ALFs, and LTCFs can differ markedly by sociodemographic factors, functional capacities, and care needs, they are alike in many important ways. First, those who live *within* a shared setting are often similar in terms of socioeconomic status, life experiences, and functional abilities. Although people draw comfort and support from proximity and interaction with similar others, the tendency toward identification also extends to a peer's misfortune, thus enhancing feelings of vulnerability [4]. Second, regardless of the factors precipitating relocation, moves commonly co-occur with loss of function, social status, a spouse, a home, prized possessions, or a community [8],[9]. Transition also involves psychological adjustment, often manifesting as anxiety, confusion, fear, helplessness, hopelessness, indecisiveness, loneliness, suicidal thoughts, and suspicion [10],[11]. The most severe relocation effects usually occur immediately after the move [12] and may persist.

### Suicide in Older Adults

Although adults over age 65 compose 12.4% of the U.S. population, they account for 14% of all suicides [13]. In 2006 the suicide rate in older adults was 14.2 per 100,000. Suicide rates among older adults vary by age, sex, and race, and are highest among white men over age 85. Methods of suicide also vary by sex. Women are more likely to use suffocation or poisoning, including medications, whereas men are more likely to use firearms. About 75% of older adults who die by suicide have never made a prior attempt [14], but because they tend to use more lethal means, they are more likely to die in an attempt [15].

Risk factors for suicide in older adults have been defined using the retrospective “psychological autopsy” [16]. The psychological autopsy is a research method in which mental and physical health status and social circumstances of the decedent are reconstructed from records and interviews with next of kin and other knowledgeable informants. These studies consistently find a close association between suicide and late life depression. Other risk factors include anxiety, substance use, and primary psychotic disorders; social dependency or isolation; family discord; losses; personality inflexibility and rigid coping style; substance misuse; and access to firearms. Since seniors with failing health are most likely to move into congregate settings, the significant relationship between late life suicide and pain, physical illness, and role function decline are of particular note [16]. A study in Canada linking prescription records with coroner's reports found that the relative risk for suicide increased with the number of physical illnesses [17].

### Suicide in Residential Communities

There are few published reports on suicide in ILCs and ALFs. Predictors of suicide identified within a cohort of 11,888 retirement community residents included pessimistic mental outlook; being widowed or divorced; sleeping ≥9 hours per night; and drinking ≥3 alcoholic beverages daily [18].

In a retrospective study of completed suicides by older adults in Finland over the course of one year (N = 1,397), NH residents constituted 0.9% [19]. Using psychological autopsies investigators found a diagnosable psychiatric illness in each case; three-quarters of those who committed suicide had depressive disorders, yet symptoms were recognized in only a third of cases. In a study of 298 Italian LTCFs with a combined resident population of approximately 28,000, investigators reported five suicide deaths (18.6/100,000) and eight nonfatal suicide attempts (29.7/100,000) [20]. Eleven of the 13 residents with suicidal behavior had a history of mental disorders, and seven had lived in the NH for less than one year.

In a study of suicide conducted between 1990 and 2005 in New York City residents aged over 60, investigators reported 1,771 suicides: 47 occurred in LTCFs and 1,724 in the community [21]. Residents in LTCFs who died by suicide were older than community-dwelling elders who died by suicide but the two groups did not differ by race or sex . Suicides in LTCFs were less likely to involve firearms and over 2.5 times as likely to involve jumping from heights. Over the 15-year period there was a significant decrease in the relative rate of suicide among community elders but no rate change in the LTCF population.

In a study of 1,080 LTCFs, characteristics found to be associated with increased suicidal behaviors included high staff turnover and larger facilities [22]. Passive self-harming behaviors—including self-neglecting behaviors such as refusal of medication, fluid, or food—are of concern, particularly in LTCFs, and have been implicated in increased mortality risk [22]. Because these behaviors may have less imminent death implications, however, they can overlap with or be distinct from behaviors in residents with more direct suicidal intent [23].

Predictors of depression among ILC residents included being older, having chronic health conditions, grieving a loss, socializing less, and attending church less [24]. In ALFs, more depressive symptoms were seen among older residents with greater functional impairment, poorer self-rated health, lower sense of mastery, less religiosity, and negative attitudes toward aging [25]. Estimates of depression rates in LTCFs ranged from 22% at admission [26] to over 40% [27]. Depression in LTCF residents was often undetected or untreated [27]. These studies suggest that depression is prevalent and that predictors of suicide in congregate settings are similar to those found in the community.

## Creating Healthy Communities

Suicide is often perceived as an impulsive, random act, and its precipitants are often circumstances with which other residents can relate and may even consider a normal part of aging. Current research, however, suggests an alternative view—that suicidal behavior is rooted in a culmination of factors and experiences over time [28], and that it is often preventable by addressing these underlying causes. Tenets common among the prominent developmental theories of aging—disengagement, activity, selectivity, and continuity (see Box 1)—include inevitable losses and the challenge for elders to alter cognitions and behaviors in order to accommodate age-related changes. Thus, the public health approach to suicide is consistent with theories of aging in that it calls for actions that aim to mitigate the multiple, cumulative losses for which older adults are at increased risk.

**Box 1. Developmental Theories of Aging**

- **Disengagement.** Views aging as a process of mutual withdrawal in which older adults voluntarily slow down by retiring, as expected by society. This is the idea that separation of older people from active roles in society is normal and appropriate, and benefits both society and older individuals [30].
- **Activity.** Sees a positive correlation between keeping active and aging well. This theory implies that the more active elderly people are, the more likely they are to be satisfied with life [30].
- **Selectivity.** Suggests that older people may benefit from becoming more active in some aspects of their lives, more disengaged in others. Thus older persons do not simply react to social contexts but proactively manage their social worlds [31].
- **Continuity.** States that older adults will usually maintain the same activities, behaviors, personalities, and relationships as they did in their earlier years of life. Older adults try to maintain this continuity of lifestyle by adapting strategies that are connected to their past experiences [30].

Suicide prevention approaches can be classified broadly as targeting either the whole population or those at more immediate risk. Table 1 outlines a set of goals and objectives that can be pursued in each category through an array of possible programs and policies. While these strategies per se are not evidence-based, they address risk and protective factors that have been empirically linked to the incidence of suicidal behavior.

**Table 1:** Opportunities for suicide prevention interventions in senior living communities.

Approach	Goal	Objective
**At-risk approaches**	**Increase help-seeking behaviors**	• Increase residents' knowledge of treatable risk factors, potential treatments, and available services• Address local barriers to help-seeking• Implement efforts to reduce stigma and normalize help-seeking
	**Identify and refer distressed or at-risk residents**	• Increase the ability of other residents, staff, and families to identify and refer residents for help (i.e., “gatekeeper training”)• Increase case identification of depression, substance abuse, and suicidality (i.e., screening)• Increase clinicians' capacity to identify and refer appropriately
	**Increase access to mental health and substance abuse services**	• Create linkages with community-based mental health and substance abuse services• Provide mental health and substance abuse services or supports
	**Promote effective treatment and management of mental health and substance abuse disorders**	• Adhere to geriatric-specific treatment guidelines• Utilize effective models of geriatric care management• Assess for suicidality• Increase regular monitoring of at-risk residents
	**Effectively address medical conditions and pain**	• Employ treatment regimens designed to reduce symptoms and pain• Help ill residents deal with specific types of disability and functional impairment
**Whole-population approaches**	**Promote effective coping and functioning**	• Promote coping with loss, bereavement• Promote coping with decreased functioning, role changes• Promote problem-solving skills• Provide assistance with financial or other matters
	**Promote social networks and social support**	• Encourage connection among residents• Promote a sense of community on campus• Provide or facilitate regular “check-ins”• Facilitate contacts with family members
	**Promote engagement in positive activities**	• Provide access to spiritual or faith activities• Promote involvement in volunteer activities• Provide recreational activities• Promote engagement in physical activity
	**Decrease access to lethal means**	• Restrict access to firearms• Limit access and/or erect fences on roofs of buildings• Replace windows or limit size of window openings• Restrict access to stored chemicals and prescription drugs

Source: *Report on the 2008 Summit on Opportunities for Mental Health Promotion and Suicide Prevention in Senior Living Communities* (2009) [29].

### At-Risk Approaches

Such approaches focus on identifying and assisting residents who are suicidal, have symptomatic mental health problems, or are at higher risk for suicide. These “at-risk strategies” will likely involve both instituting facility-specific practices and working collaboratively with outside service providers.

Given the high risk for suicide associated with mood disorders in older adults, the detection and effective management of psychiatric illness is a high priority. Elements of this approach may include using screening tools to detect depression or suicidal ideation and facilitating ready access to mental health and substance abuse expertise, either within the facility or by referral to affiliated providers. Similarly, given the associations among medical illness, functional impairment, pain, and suicide in older adults, aggressive management of these conditions can be viewed as a critical component of a suicide prevention program. It is also important to normalize and support help-seeking by residents. Because residents themselves are in a position to notice behaviors of concern in peers, strategies to impart knowledge and skills regarding appropriate interventions are worth consideration.

### Whole Population Approaches

The group nature of residential care renders such communities ideal for population-based approaches to suicide prevention. The major features of a population approach include reducing risk factors, increasing protective factors, and creating health-promoting environments yielding benefits for all regardless of risk status. In the context of residential care, whole population approaches might include promoting health and supporting older adults as they manage relocation stresses throughout the adjustment period (Table 1). Because social isolation and interpersonal discord place older adults at risk for suicide, and because supportive social networks appear to mitigate risk, the promotion of supportive social communities is critical, along with systematic efforts to engage residents in positive activities. Although physical activity has not been linked specifically to reduced risk, its association with emotional well being and maintenance of function indicate its potential role as a suicide preventive intervention. Finally, restricting access to lethal means for taking one's life should be considered, certainly for the older person with depression (e.g., removing a firearm from the home as an at-risk approach) but also as a component of community-wide safety. Facilities can scan their environments for potential means (e.g., access to rooftops, unsecured medications) and take measures to reduce their accessibility.

## Initial Steps toward Suicide Prevention Planning

It is important for health care providers and administrators who work in senior living communities to acknowledge that, despite their vigilance in providing safe, supervised environments, the populations they serve have underlying risk factors that predispose them to distress and possibly suicidal behaviors. While suicides may be uncommon, a single event can negatively affect the community in significant ways.

A first step toward changing the attitudes and practices of those who live and work in these environments is increased attention to the potential for suicide and consideration of how existing policies, procedures, and programs may potentially mitigate or exacerbate risk. Because the functional capacities of residents, health care resources, quality indicators, and financial incentive structures differ by setting, the intensity of need and opportunities for intervention vary. There is no single blueprint for a suicide prevention plan. It is incumbent upon each facility to assess its own characteristics and resident populations and to use that information to set priorities and establish relevant goals. Some special considerations are presented in Box 2.

**Box 2. Special Considerations for Senior Living Facilities in Developing Suicide Prevention Plans and Creating Healthy Communities**

- Does the facility accommodate residents with special needs (e.g., cognitive impairment, sensory deficits, functional limitations) by providing equal access to recreational activities and social events, and accessibility throughout the facility and grounds?
- Does the facility's location (e.g., rural/urban; co-located on a campus; proximity to water or highways) provide unique circumstances that could promote active engagement or pose safety risks?
- Is the facility located in a community with resources that could augment programs offered by the facility (e.g., HMO-offered disease self-management groups; disease-specific not-for-profit organizations that offer support groups, self-help groups, informational sessions, academic institutions)?
- Does the facility provide or have access to knowledgeable service coordinators, health care providers trained in geriatrics, mental health services, substance abuse programs, and emergency services?
- Does the facility offer programs, activities, and services that match the personal and cultural preferences of its residents (e.g., physical activity, religious/spiritual, individual/group)?
- Does the facility have policies and procedures that weigh the resident's right to privacy with the safety of the resident and community (e.g., access to firearms, alcohol, medications)?
- Has the facility considered all that would be involved in screening for depression and/or suicide (e.g., residents with cognitive impairment; non-English–speaking residents; linkages with providers for residents who screen positive; policies and procedures for assisting residents who screen positive; regulatory incentives/disincentives for identifying residents with depression)?

Finally, facilities should consider developing policies and procedures in order to be prepared in the event that a suicide attempt or death does occur. Advance planning should include protocols for follow-up care after an attempt to address how the facility will work with the media; communicate about a suicide death to the community; and provide support for those most affected by the suicide.

## Abbreviations

ADL: activities of daily living
ALF: assisted living facility
ILC: independent living community
LTCF: long-term care facility
NH: nursing home
